# Barriers and facilitators of patient centered care for immigrant and refugee women: a scoping review

**DOI:** 10.1186/s12889-020-09159-6

**Published:** 2020-06-26

**Authors:** Tali Filler, Bismah Jameel, Anna R. Gagliardi

**Affiliations:** Toronto General Hospital Research Institute, University Health Network, 200 Elizabeth Street, 13EN-228, Toronto, ON M5G2C4 Canada

**Keywords:** Patient-centred care, Migrants, Immigrants, Refugees, women’s health, Healthcare inequities, Barriers, Facilitators, Scoping review

## Abstract

**Background:**

Migrants experience disparities in healthcare quality, in particular women migrants. Despite international calls to improve healthcare quality for migrants, little research has addressed this problem. Patient-centred care (PCC) is a proven approach for improving patient experiences and outcomes. This study reviewed published research on PCC for migrants.

**Methods:**

We conducted a scoping review by searching MEDLINE, CINAHL, SCOPUS, EMBASE and the Cochrane Library for English-language qualitative or quantitative studies published from 2010 to June 2019 for studies that assessed PCC for adult immigrants or refugees. We tabulated study characteristics and findings, and mapped findings to a 6-domain PCC framework.

**Results:**

We identified 581 unique studies, excluded 538 titles/abstracts, and included 16 of 43 full-text articles reviewed. Most (87.5%) studies were qualitative involving a median of 22 participants (range 10–60). Eight (50.0%) studies involved clinicians only, 6 (37.5%) patients only, and 2 (12.5%) both patients and clinicians. Studies pertained to migrants from 19 countries of origin. No studies evaluated strategies or interventions aimed at either migrants or clinicians to improve PCC. Eleven (68.8%) studies reported barriers of PCC at the patient (i.e. language), clinician (i.e. lack of training) and organization/system level (i.e. lack of interpreters). Ten (62.5%) studies reported facilitators, largely at the clinician level (i.e. establish rapport, take extra time to communicate). Five (31.3%) studies focused on women, thus we identified few barriers (i.e. clinicians dismissed their concerns) and facilitators (i.e. women clinicians) specific to PCC for migrant women. Mapping of facilitators to the PCC framework revealed that most pertained to 2 domains: fostering a healing relationship and exchanging information. Few facilitators mapped to the remaining 4 domains: address emotions/concerns, manage uncertainty, make decisions, and enable self-management.

**Conclusions:**

While few studies were included, they revealed numerous barriers of PCC at the patient, clinician and organization/system level for immigrants and refugees from a wide range of countries of origin. The few facilitators identified pertained largely to 2 PCC domains, thereby identifying gaps in knowledge of how to achieve PCC in 4 domains, and an overall paucity of knowledge on how to achieve PCC for migrant women.

## Background

The rate of both voluntary (immigrants move for better opportunities in another country) and involuntary (refugees move to escape dangerous conditions in their home country) migration has been steadily rising [1]. From 2000 to 2017, the total number of international migrants rose from 173 million to 258 million, an increase of 49% [2]. Research shows that migrants are less likely than the general population to experience high-quality health care [3]. For example, a systematic review (67 studies, 1996–2009) of population-based studies involving immigrants in the United States found they were less likely to have medical insurance, or access to a regular healthcare provider, preventive care, tests or services; and were more likely to report insufficient time with clinicians and not being engaged by clinicians [4]. Interviews with immigrants of various ethnic origins in the Netherlands [5], and with Asian immigrants in the United States [6] revealed they had experienced negative health care events, described as abusive or discriminatory and potentially dangerous, due to language barriers and cultural differences. Similarly, a scoping review (27 studies, 1993–2014) of studies based in Canada involving immigrants from various ethnic origins found that access to and quality of primary care was influenced by communication and cultural factors [7].

Several organizations have advocated for action to improve the health of immigrants and refugees. For example, the University of Edinburgh, the European Public Health Association and NHS Health Scotland hosted the First World Congress on Migration, Ethnicity, Race and Health in May 2018 to explore how to improve the quality of care for migrants [8]. A scoping review (83 studies, 1990–2015) of interventions used to improve the health of migrants conducted by The Worldwide Universities Network’s Health Outcomes of Migration Events research group revealed that all interventions aimed to educate migrants to prevent or self-manage conditions such as diabetes and cardiovascular disease [9]. The authors concluded that more research was needed to fully investigate factors influencing quality of care for a broader range of conditions as the first step in developing interventions to improve the organization, delivery and outcomes of health services for migrants. The World Health Organization (WHO), in collaboration with the United Nations and the International Organization for Migration, generated a framework of priorities to promote the health of immigrants and refugees [2]. The WHO Global Action Plan emphasizes the need to improve the quality, acceptability, availability and accessibility of health care services for migrants.

The WHO Global Action Plan makes special mention of improving the health and well-being of women given considerable evidence of persistent gendered inequities in health care quality in both lower- and higher-resourced countries [10–12]. For example, immigrant women have experienced poor access to breast and cervical cancer screening [13], dissatisfaction with health care experiences for maternity [14], contraceptive counseling [15], and menopause [16], and may be uncomfortable with physical exams even when performed by a woman physician [7]. A scoping review (29 studies, 1995–2016) of interventions to reduce adverse health outcomes resulting from gender bias among immigrant populations revealed that most studies focused on counseling or education on domestic violence among Latino populations in the United States [17]. Clearly, more research is needed on how to improve quality of care for migrant women for the range of health issues and populations.

The concept of cultural competence has emerged in response to widespread disparities in care by culture, race, ethnicity, religion, gender and sexual orientation, and refers to care that respects patients’ health beliefs about their illness and its causes, interprets health issues from a biopsychosocial rather than biomedical context, involves communication in language accessible to patients, and engages patients in developing a mutually agreeable treatment plan [18]. Models or frameworks of cultural competence emphasize the need for clinicians to be culturally competent, referring to understanding and respecting cultural differences, but otherwise provide limited guidance on approaches or processes to practice cultural competence at the point of care [19]. Culturally competent care and patient-centred care (PCC) share many of the same principles and both aim to tailor care to individual patients, yet considerably more research has explored determinants and impacts of PCC [18]. PCC is a multi-dimensional approach whereby clinicians foster a healing relationship, exchange information, respond to emotions, manage uncertainty, engage patients in decisions, and enable self-management, and in so-doing, tailor care to an individual’s clinical needs, life circumstances, and personal values and preferences, all of which may be influenced by culture, race, ethnicity, religion, gender and sexual orientation [20]. Moreover, PCC has been associated with a range of beneficial patient-important and clinical outcomes [21]. PCC is one way to reduce gendered disparities in health care quality among immigrant and refugee women [17]. The purpose of this study was to review published research on determinants (barriers, facilitators) or approaches of PCC specifically for immigrant and refugee women. This knowledge could be used to design and evaluate strategies or interventions that improve migrant women’s health care experiences and outcomes.

## Methods

### Approach

We conducted a scoping review [22, 23], and complied with the Preferred Reporting Items for Systematic Reviews and Meta-Analysis criteria for scoping reviews (PRISMA-Scr) [24]. While similar in rigour to traditional systematic reviews, scoping reviews include studies with a range of research designs; focus on characterizing the literature to describe the nature of existing knowledge and identify issues for which further primary research is needed; and do not assess the methodological quality of included studies [22, 23]. A scoping review is comprised of five steps: scoping, searching, screening, data extraction and data analysis [22, 23]. We did not require research ethics board approval as data were publicly available, and we did not register a protocol.

### Scoping

The scoping step involved becoming familiar with the literature on this topic. We conducted a preliminary search in MEDLINE using Medical Subject Headings: “emigrants and immigrants” or “refugees” and “patient-centered care”. Two research assistants, TF and BJ, with guidance from ARG, screened titles and abstracts of the search results to identify examples of relevant studies. We used this insight to develop eligibility criteria and generate a more detailed search strategy.

### Eligibility criteria

We drafted eligibility criteria according to the Population, Issues, Comparisons and Outcomes (PICO) framework. *Population* referred to immigrant or refugee adults aged 18 or older with any health issue in any setting of care (i.e. primary, hospital) or country. We did not restrict eligible studies to women only participants, as studies with both women and men might report sub-analyses by sex or gender. As many authors do not distinguish sex (female/male biological attributes) and gender (socially-constructed roles, behaviours and identities), we reported results for women, and defined women as individuals who self-identified as women or were identified as such by authors. We also included studies where participants were clinicians (physicians, nurses), as such research might aim to reveal determinants or approaches of PCC for migrant women. The *intervention* of interest included barriers, facilitators, approaches, strategies, programs or tools used to promote or support PCC by influencing patient and/or clinician awareness, knowledge, self-efficacy, attitude, adoption or implementation of PCC for immigrants or refugees. We defined PCC as partnership between clinicians and patients (also family, care partners) to discuss and tailor care according to individual needs and characteristics [20]. To be eligible, the article had to employ the term “patient-centred” or a synonymous term (i.e. person-centred, client-centred) or variant spelling of these terms, or be indexed with the Medical Subject Heading “patient-centered care”. *Comparisons* referred to studies that explored or compared patient and/or clinician views about what constitutes PCC or experiences of PCC, described approaches desired or employed to achieve PCC (evaluated alone, before-after the intervention, or in comparison with another intervention), identified determinants (facilitators, barriers) of PCC, or evaluated the impact of interventions designed to promote or support PCC. *Outcomes* included any reported by eligible studies including but not limited to: awareness, understanding, experiences or impacts of PCC; elements of PCC; patient engagement in or satisfaction with care; relationship between the patient and clinicians; or the influence on health outcomes as a result of the above factors. Eligible study designs included qualitative (interviews, focus groups, qualitative case studies), quantitative (questionnaires, randomized controlled trials, time series, before/after studies, prospective or retrospective cohort studies, case control studies) or mixed-methods studies published in English-language in peer-reviewed journals. We included studies published from 2010, when the concepts of cultural competency and patient-centred care became prominent [18], to current.

We excluded studies in which the setting was long-term care or the patient-centred medical home; the population was family or care partners only, or allied healthcare professionals; or the intervention pertained to the illness experience rather than the care experience, views about the treatment modality rather than the care experience; or patient engagement in research or health system planning. Protocols, editorials, commentaries, letters, news items, or meeting abstracts or proceedings were not eligible. We did not include systematic reviews, but screened references for eligible primary studies.

### Searching

The search strategy (Additional File 1) was developed by ARG, trained as a medical librarian, and complied with the Peer Review of Electronic Search Strategy reporting guidelines [25]. We searched MEDLINE, CINAHL, SCOPUS, EMBASE and the Cochrane Library on June 5, 2019. We did not include the term “woma(e)n” or “female” in the search strategy, choosing instead to to search for all studies of any migrant and PCC, as this might have reduced the number of studies retrieved by eliminating studies involving both men and women that were not also indexed by “woma(e)n” or “female”.

### Screening

To pilot test screening, TF, BJ and ARG independently screened titles and abstracts for the first 25 search results against eligibility criteria, and discussed discrepancies, and how to interpret and apply the eligibility criteria. Thereafter, TF and BJ independently screened all remaining titles and abstracts, and ARG resolved discrepancies or uncertainties. TF and BJ retrieved full-text items, which they screened concurrent with data extraction.

### Data extraction

We developed a data extraction form to collect information on author, publication year, country, study objective, research design including participant characteristics (immigrant or refugee, country of origin, clinician specialty), clinical topic, intervention or aspect of PCC studied, and results. To pilot test data extraction, TF, BJ and ARG independently extracted data from two articles, and compared and discussed findings to refine the data extraction form and the approach to data extraction. Thereafter, TF and BJ independently extracted data from all articles, and ARG resolved discrepancies or uncertainties, and independently checked completed data tables. We did not assess study quality as this is not required in a scoping review [22, 23].

### Data analysis

We used summary statistics to report study characteristics (date published, country, research design, number and type of participants, type of migrant, country of origin, and whether findings were specific to women), and clinical topic. We summarized facilitators and barriers of PCC in tabular format and text by level (patient, clinician, organization/system), type of study participant (patients, clinicians) and those that pertained specifically to care for women. To further characterize facilitators that emerged from included studies, we mapped them to an established framework of PCC, chosen because it was rigorously developed and comprehensive, comprised of 31 elements organized in 6 domains: foster a healing relationship, exchange information, respond to emotions, manage uncertainty, make decisions, and enable self-management [20]. We then summarized the number and type of PCC domains addressed by included studies for migrants in general, and for women migrants.

## Results

### Search results

**Fig. 1:**
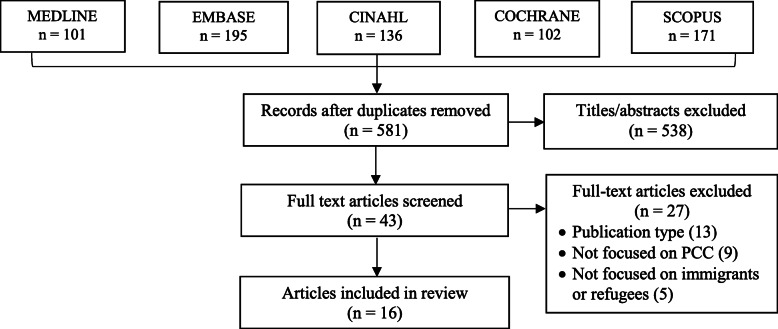
PRISMA diagram. Flow chart of studies identified, screened and included

A total of 581 unique articles were identified, and 538 were excluded upon screening of titles and abstracts. Among 43 full-text articles that were screened, 27 were excluded because they were not an eligible publication type (13), not focused on PCC (9) or not focused on immigrants or refugees (5). We did not identify additional items in the references of eligible studies. A total of 16 studies were eligible for review (Fig. 1). Data extracted from included studies are available in Additional File 2 [26–41].

### Study characteristics

Study characteristics are summarized in Table 1. Studies were published between 2010 and 2019 (50.0% in last 3 years). Studies were conducted in the United States (6), Australia (3), Canada (2), Netherlands (2), Sweden (2) and Norway (1). Most (14, 87.5%) studies were qualitative (interviews, focus groups), and 2 (12.5%) employed a questionnaire. Qualitative studies involved a median of 22 participants (range 10 to 60). One survey included 107 participants, and the other survey included 598 participants. Eight (50.0%) studies involved clinicians only, 6 (37.5%) patients only, and 2 (12.5%) both patients and clinicians.

**Table 1:** Characteristics of included studies

Study	Objective	Design	Clinical topic	Participants (n)	Migrant type	Migrant origin	Specific to women (% women)
Harding [26] 2019 Australia	Barriers	Interviews	General	Clinicians (14)	Refugees	Syria, Iraq, Sudan, Burma	No
Winn [27] 2018 Canada	Barriers, facilitators	Interviews	Maternity	Clinicians (10)	Refugees	Syria, Iraq, Eritrea, Congo, Afghanistan	Yes
Mollah [28] 2018 Australia	Barriers, facilitators	Interviews	Mental health	Clinicians (20)	Immigrants and refugees	General	No
Murray [29] 2018 Australia	Facilitators	Interviews	Medication	Patients (17) Clinicians (13)	Refugees	Bhutan	No (17.6)
Hjorleifss [30] 2018 Norway	Facilitators	Focus groups	General	Clinicians (28)	Immigrants	Asia, South America, Europe	No
Jones [31] 2018 United States	Barriers, facilitators	Interviews	General	Patients (20)	Immigrants	Mexico	No (65.0)
Mohammadi [32] 2017 Sweden	Barriers	Interviews	Maternity	Patients (11)	Refugees	Afghanistan	Yes
Paternotte [33] 2017 Netherlands	Facilitators	Interviews	General	Patients (30)	NR (“non-native”)	Surinam, Turkey, Morocco, Portugal, Indonesia, Iraq, China, Ireland, United States	No (not reported)
Larsson [34] 2016 Sweden	Barriers	Interviews	Abortion	Clinicians (13)	Immigrants	General	Yes
Paternotte [35] 2016 Netherlands	Barriers	Interviews	General	Clinicians (17)	NR (“non-native”)	Morocco, Turkey, Hungary, Nicaragua, Australia, Belgium, Pakistan, Nigeria	No
Phillippi [36] 2016 United States	Facilitators	Interviews	Maternity	Patients (50)	Immigrants	Cambodia, Somalia, Syria, Iraq, Burma, Mexico, South America	Yes
Clochesy [37] 2015 United States	Barriers	Focus groups	General	Patients (60)	Immigrants (31.7%)	Mexico, South America, Russia	No (46.7)
De Jesus [38] 2014 United States	Facilitators	Focus groups	Mental health	Patients (48)	Immigrants	Brazil, Cape Verde (Portuguese)	No (50.0)
Papic [39] 2012 Canada	Barriers, facilitators	Survey	General	Clinicians (598)	Immigrants	General	No
Hasnain [40] 2011 United States	Barriers, facilitators	Survey	General	Patients (27) Clinicians (80)	Immigrants	General (Muslim)	Yes
Lo [41] 2010 United States	Barriers	Interviews	General	Clinicians (24)	Immigrants	General	No

A total of 9 (56.3%) studies pertained to care in general, while others focused on family planning or maternity care (4, 25.0%), mental health care (2, 12.5%), and medication management (1, 6.3%). All studies explored facilitators or barriers of care for immigrants or refugees. No studies developed or evaluated strategies, interventions or tools aimed at either migrants or clinicians to improve quality of care.

Most studies were specific to immigrants (9, 56.3%), while 4 (25.0%) were specific to refugees, 2 (12.5%) referred to “non-natives”, and 1 (12.5%) study involved both immigrants and refugees. Among the 8 studies involving patients or patients and clinicians, all noted the country of origin (Afghanistan, Bhutan, Brazil, Burma, Cambodia, Cape Verde, China, Indonesia, Iraq, Ireland, Mexico, Morocco, Portugal, Russia, Somalia, South America, Surinam, Syria, Turkey, United States) or ethnicity/culture (Portuguese, Muslim) of patients. Of the remaining 8 studies involving clinicians only, 4 (50.0%) pertained to immigrants or refugees in general, and 4 (50.0%) pertained to specific groups (Afghanistan, Asia, Australia, Belgium, Burma, Eritrea, Europe, Democratic Republic of Congo, Hungary, Iraq, Morocco, Nicaragua, Nigeria, Pakistan, South America, Sudan, Syria, Turkey).

### Barriers of caring for migrants

Eleven (68.8%) studies reported barriers of caring for immigrants/refugees [26–28, 31, 32, 34, 35, 37–40]. Table 2 summarizes barriers by level (patient, clinician, organization or system) and who articulated the barrier (patient, clinician, both). At the patient level, both patients and clinicians viewed language as a patient-level barrier. Clinicians thought that culture influenced patient views about health and illness, expectations of clinicians or the healthcare system, and acceptance of or adherence to procedures or treatment. Patients identified few patient-level barriers of care.

**Table 2:** Barriers of PCC for immigrants and refugees

Level	Articulated by (occurrences across included studies if > 1)		
	Patient	Clinician	Both
Patient	**All patients** • Feel vulnerable when they need help • Reluctant to “bother” nurse to ask for help **Women patients** none	**All patients** • Culture influences expectations of healthcare provider or system and views about illness, i.e. shame about condition (4) • Acceptance of procedures or treatment/adherence (4) • Diversity of cultures/languages requiring some familiarity • Lack of familiarity with healthcare system **Women patients** • Little knowledge about disease processes • Little knowledge about female anatomy, menstrual cycle, reproduction, contraceptives • Culture/religion influences contraceptive decisions, leading to unplanned pregnancy/abortion • Fear of violence if families learn about contraceptive use, pregnancy or abortion	**All patients** • Language (5) **Women patients** • Decisions made by family rather than the individual woman (2) • Economic constraints or lack of health insurance (2) • Lack of trust in health care system; sometimes due to past negative experience (2)
Clinician	**All patients** • Busy and rushed, so little communication (2) • Delayed diagnosis (2) • Treated like a lab rat rather than a person; wanted clinicians to get to know them, listen, care, help them understand • Judgmental behavior or tone • Treated differently due to culture, race, gender **Women patients** • Ignored/dismissed concerns • Provided little information about possible complications or about actual adverse outcomes • Disrespectful behavior or disparaging remarks	**All patients** • Lack of training in cultural competency or how culture influences communication or health (seeking) behavior (4) • How to achieve cultural competency without stereotyping (2) • How to deliver care while accommodating culture (2) • Unaccustomed to managing certain diseases/health care issues (i.e. trauma, mental health, tuberculosis) • Anxiety due to lack of knowledge or experience with migrants • Burnout • Perceived that patients wanted doctor to lead the conversation **Women patients** none	**All patients** • Consultations require longer time due to language, culture, knowledge barriers; relationships took longer to establish (5) **Women patients** • Lack of knowledge about culture/religion
Organization or system	**All patients** • Red tape/paperwork • System difficult to navigate **Women patients** none	**All patients** • Lack of language services; reliance on family (2) • Interpreters are time-consuming and inaccurate (2) • Using family interpreters raises privacy and ethics issues (2) • Remuneration insufficient for time required (2) • Lack of support/community services • Western healthcare model inflexible **Women patients** • No protocols or guidelines to help care for migrant women	**All patients** none **Women patients** none

At the clinician level, patients and clinicians agreed that language, culture and knowledge barriers resulted in longer consultations. Clinicians noted they lacked training in cultural competency. They also said it was challenging to be culturally competent without stereotyping, and to deliver medical care while accommodating culture. Patients said that clinicians were busy and rushed, leaving little time for communication, resulting in delayed diagnoses.

At the organization or system level, clinicians felt that remuneration was insufficient for the additional time required to care for immigrants or refugees. They also noted a lack of language services, or that interpreters were inaccurate and using them was time-consuming. Instead, they relied on family members to interpret, but recognized privacy and ethical issues of doing so. Patients identified few barriers at the organization or system level.

### Facilitators of caring for migrants

Ten (62.5%) studies reported facilitators of caring for immigrants/refugees [27–31, 33, 36, 38–40]. Table 3 summarizes facilitators by level (patient, clinician, organization or system) and who articulated the barrier (patient, clinician, both). Neither patients nor clinicians identified patient-level facilitators.

**Table 3:** Facilitators of PCC for immigrants and refugees

Level	Articulated by (occurrences across included studies if > 1)		
	Patient	Clinician	Both
Patient	**All patients** None	**All patients** None	**All patients** none
	**Women patients** none	**Women patients** none	**Women patients** Communication skills/style
Clinician	**All patients** • Listen to patient; focus attention on them, not computer • Ask questions to fully understand patient’s concern • Acknowledge concerns • Offer comfort and encouragement • Prepare ahead of time • Be honest about diagnosis • Treat patient as person and not a disease **Women patients** • Time to ask questions • Lack of judgment • Being provided with information so they could be involved in decisions • Perceived clinical competency	**All patients** • Coordinate tests and appointments (2) • Personal dedication (2) • Devote more time to consultations or divide tasks into multiple consultations (2) • Self-awareness of the influence of one’s own culture • Take time to describe how the healthcare system works • Ensure the patient accepts use of an interpreter • Learn a few words of patient’s language **Women patients** none	**All patients** • Establish rapport: greet and welcome the patient, take time to chat informally, adopt a friendly, caring and respectful manner (7) • Clear communication: speak slowly, use short sentences, explain topics in various ways, avoid medical jargon (4) • Doctor of same culture or gender, or of older age (3) • Take extra time to ensure/check comprehension (3) • Recognize/accommodate/respect cultural differences (3) • Become familiar with patient’s culture and migration journey (3) • Involve personal support network as interpreters (2) • Use skilled interpreters rather than family (2) • Use verbal and audiovisual rather than written communication (may lack literacy even in own written language (2) • Personalize care, don’t generalize to culture or country of origin **Women patients** • Gender (2) • Communication skills • Ethnicity/religion
Organization or system	**All patients** none **Women patients** none	**All patients** • Collaboration with community agencies • Promote a culture of diversity • Access to language services (2) **Women patients** none	**All patients** • Offer orientation to or tours of healthcare services (4) • Multidisciplinary teamwork (3) • Continuity of health care team **Women patients** none

At the clinician level, both patients and clinicians identified numerous facilitators. Most frequently, they recommended establishing rapport by greeting and welcoming the patient, taking time to chat informally, and adopting a friendly, caring and respectful manner. Other facilitators suggested by both patients and clinicians included clear communication (speak slowly, use short sentences, explain topics in various ways, avoid medical jargon, employ audiovisual rather than print information), take extra time to check comprehension, become familiar with the individual patient’s culture and migration journey, accommodate and respect cultural differences. Some patients and clinicians preferred skilled interpreters while others preferred family members to assist with communication. Patients and clinicians also viewed doctors of the same culture or gender as a facilitator. Clinician-level facilitators proposed by clinicians included booking longer consultations or dividing tasks into multiple consultations, coordinating internal and external appointments, and personal desire or dedication to help immigrants and refugees. Clinician-level facilitators suggested by patients included listening to patients, asking questions, acknowledging concerns, and treating the patient as a person and not a disease.

At the organization or system level, study participants identified few facilitators. Both patients and clinicians recommended orientation sessions or tours of health care facilities or systems, and multidisciplinary teamwork. Clinicians recommended access to language services and partnerships with community agencies. No patients identified organizational or system level facilitators.

### Barriers and facilitators of caring for women migrants

Five (31.3%) of 16 included studies focused on women. Of those, 2 (40.0%), involved women as participants [32, 36], 2 (40.0%) involved clinicians as participants [27, 34], and 1 (20.0%) study included both [40]. Among the 5 women-focused studies, 4 (80.0%) pertained to family planning or maternity care [27, 32, 34, 36], and 1 (25.0%) focused on the general care of Muslim women [40]. Other studies did not explore issues specific to women or report sub-analyses by gender. Barriers and facilitators of caring for women immigrants/refugees are summarized in Tables 2 and 3, respectively.

Barriers noted by both women and clinicians at the patient-level included decision-making by the family rather than the individual woman, economic constraints limiting access to care, and lack of trust in the healthcare system. Clinicians noted that patient-level barriers among women included little knowledge about disease processes, female anatomy, reproduction or contraceptives; the influence of culture or religion on contraceptive decisions leading to unplanned pregnancy and abortion; and that women feared family violence if contraceptive use, pregnancy or abortion were discovered. No women identified patient-level barriers. Both women and clinicians noted that lack of knowledge about culture or religion was a clinician-level barrier. Clinicians did not identify clinician-level barriers. Women said that clinicians ignored or dismissed their concerns, provided little information about potential complications or the reason for adverse outcomes, and behaved disrespectfully or made disparaging remarks. At the organization or system level, only clinicians identified a barrier: lack of guidelines to help them care for immigrant or refugee women.

With respect to facilitators, both women and clinicians noted that patient communication skills or style was a facilitator. No other patient-level facilitators were identified by women or clinicians. At the clinician level, both women and clinicians said that clinician communication skills, gender and ethnicity or religion were facilitators. No clinicians identified clinician-level facilitators. Women recommended that clinicians take time to ask questions, assume a non-judgmental tone or manner, and provide information so that women could participate in decisions. No women or clinicians identified organization or system level facilitators.

### Other comparisons

We compared barriers and facilitators articulated by patients based on type of care (Additional File 2). Views and experiences pertaining to mental health care and inpatient care were similar to those of general/primary care. With respect to maternity care, women expressed barriers and facilitators similar to general/primary care, but also noted that family influence on decisions was a barrier, and women physicians was a facilitator. We compared barriers and facilitators based on research design (Additional File 2). The majority of included studies collected data using qualitative methods. Studies that employed survey methods generated similar barriers (language and culture differences, lack of clinician training and time) and facilitators (clinician communication skills, longer appointments for discussion/questions) as did qualitative studies. We also compared barriers and facilitators articulated by patients based on migrant status (Additional File 3). There were no clear differences in barriers and facilitators described by refugees versus immigrants. In the only study of immigrant patients to explore barriers, language differences emerged as a challenge. This was confirmed by refugees, who also said that differences in culture, and lack of clinician training and time were barriers. With respect to facilitators, both refugees and immigrants recommended that clinicians establish rapport, and take the time to communicate clearly and address questions.

### Patient-centred care for migrants

Table 4 shows facilitators of PCC for migrants mapped to an established PCC framework [20]. In total, 33 facilitators were relevant to immigrants or refugees in general. While these general facilitators spanned all PCC domains, most (23, 69.7%) pertained to the 2 domains of fostering a healing relationship and exchanging information. Few pertained to the remaining 4 domains: address emotions/concerns, manage uncertainty, make decisions, and enable self-management. Only 6 facilitators were specific to women, and these mapped to 3 PCC domains: foster a healing relationship, exchange information, and make decisions.

**Table 4:** Facilitators of PCC for migrants mapped to patient-centred care domains

Patient-centred care		Immigrant and refugee target	
Domains [20]	Description	General	Women
Foster a healing relationship	Establishing a friendly, courteous and comfortable relationship	• Focus attention on the patient (not computer) • Prepare ahead of time • Treat patient as person and not a disease • Be dedicated to help migrants • Promote a culture of diversity • Be self-aware of the influence of one’s own culture • Greet and welcome the patient • Take time to chat informally • Adopt a friendly, caring and respectful manner • Doctor of same gender, culture or religion, or of older age • Become familiar with patient’s culture and migration journey • Learn a few words of patient’s language • Ensure continuity of the healthcare team	• Assume a non-judgment manner • Convey clinical competency • Woman doctor, or doctor of same culture or religion
Exchange information	Learning about the patient; words or language used to discuss health care	• Listen to the patient • Ask questions to fully understand patient’s concern • Involve personal support or trained interpreter • Ensure the patient accepts use of an interpreter • Speak slowly • Use short sentences • Avoid medical jargon • Explain topics in various ways • Devote more time to consultations or divide tasks into multiple consultations • Take extra time to check/ensure comprehension	• Provide time to ask questions • Good communication skills
Address emotions or concerns	Responding to or managing emotional reactions	• Acknowledge concerns • Offer comfort and encouragement • Apply multidisciplinary teamwork	–
Manage uncertainty	Addressing uncertainties about prognosis or outcomes	• Be honest about diagnosis	–
Make decisions	Engaging patient in discussion and decision-making	• Recognize, accommodate and respect cultural differences • Personalize care (don’t generalize to culture or country of origin)	• Provide enough information that they are equipped to take part in decisions
Enable self-management	Setting expectations for follow-up care; preparing for self-managing health and well-being	• Coordinate tests and appointments • Take time to describe how the healthcare system works or offer orientation/tours • Use verbal and audiovisual rather than written communication (may lack literacy even in own written language) • Collaborate with community agencies	–

## Discussion

This scoping review of PCC for immigrants and refugees identified few studies overall and even fewer that focused on PCC for women. Most studies focused on care in general rather than specific diseases or healthcare issues, thus PCC for migrants with specific conditions remains unknown. All studies explored barriers and/or facilitators of PCC; none evaluated interventions to improve PCC. While few studies were included, they revealed numerous barriers of PCC at the patient, clinician and organization/system level for immigrants and refugees from a wide range of countries of origin. Studies also reported facilitators of PCC, though largely at the clinician level; thus, patient and organization/system level facilitators of PCC for immigrants and refugees are not fully known. Barriers are facilitators were similar by type of migrant (refugees versus immigrants), care (general/primary, mental health, maternity), and research design (qualitative versus quantitative). Only 5 studies pertained to women, and addressed family planning or maternal care. No additional studies reported results by sex/gender. Thus few barriers or facilitators specific to PCC for immigrant or refugee women emerged. Mapping of facilitators to a PCC framework identified specific PCC domains for which knowledge of strategies to achieve PCC for migrants is lacking, particularly women migrants.

This study confirms prior research on factors that influence PCC for migrants. A scoping review (27 studies, 1993–2014) of studies based in Canada involving immigrants from various ethnic origins found that quality of primary care was influenced by patient-specific factors including culture (i.e. social stigma of disease, disrespectful to address elders by first name), communication (i.e. language skills, print information not regarded as reliable) and socioeconomic (i.e. unable to attend appointments due to multiple jobs or shift work) factors [7]. An integrative literature review (35 studies, 1997–2015) on cultural competence in cancer management identified clinician-specific factors such as skills, awareness, knowledge, and personal characteristics influenced patient-provider communication [42]. Our findings are unique from other research because we identified a greater number of determinants of high quality care for migrants; by categorizing them, we distinguished patient, clinician and organization/system level determinants, which enables targeted quality improvement efforts; by employing gender sub-analyses, we revealed aspects of care important to women migrants; and by employing a PCC lens using a framework of elements considered ideal by patients and clinicians [20], we revealed a range of approaches and processes that can be employed to improve PCC for migrants, and in particular, women migrants. Thus our study contributes many novel findings to the existing literature.

The findings, including barriers, facilitators and identified gaps in knowledge, give rise to several implications. For example, although immigrants and refugees may have differing healthcare issues and access to health services [2], this study found that immigrants and refugees articulated similar facilitators and barriers of PCC. This may suggest that strategies to implement PCC may be equally beneficial to both groups; however, given that only one study of immigrants explored barriers, further research is needed to more thoroughly compare facilitators and barriers of PCC experienced by immigrants and refugees, and whether those differ by healthcare issue, and warrant different strategies to implement PCC. Language emerged in this review as a key barrier of PCC for immigrants and refugees, and access to or the use of interpreters was the corresponding facilitator. However, the participants of included studies, both patients and clinicians, differed in preference for trained interpreters versus family interpreters, revealing pros and cons to each. As a key barrier, further research must assess strategies or interventions for overcoming language-based challenges, and likely both options are needed. In the case where trained interpreters are not available or not preferred by patients, family interpreters must be used, and research should explore how to prepare family members for this role. In the case where trained interpreters are available, but not used either because clinicians perceive them to be time-consuming or inaccurate, or because clinicians lack knowledge or skill in how to use trained interpreters, research should explore the skills and processes essential needed by interpreters to facilitate discussions with migrants, and how to train clinicians to use trained interpreters. The views and experiences of medical interpreters should also be considered [43].

This review also revealed tension or challenges in respecting and accommodating culture without stereotyping patients according to ethnicity, religion or country of origin, and without compromising medical care. A key clinician-level barrier in this study was lack of training or professional development on how to deliver culturally competent care. A Cochrane systematic review on cultural competence education for healthcare professionals (5 randomized controlled trials involving 337 clinicians and 8400 patients reflecting a variety of cultures/languages published from 1991 to 2010) found no effect on patient satisfaction with consultations, patient scores of physician cultural competency, or treatment outcomes, but patient adherence to prevention or treatment improved in the intervention group [44]. Given few studies and mixed results of the Cochrane review [44], further research is needed on how to equip clinicians to achieve PCC for immigrants or refugees, and by specific condition, as PCC may vary across health care issues.

Few studies focused on women, thus few barriers or facilitators of PCC specific to immigrant or refugee women emerged. Those that did pertained to family planning or maternity care. However, women in general experience disparities in quality of care for many health care issues across the lifespan including depression and cardiovascular disease [45, 46]. To address the WHO Global Action Plan’s call to improve the health and well-being of migrant women, further research is needed on determinants of, and interventions to support PCC for immigrant and refugee women [2]. Such research could inform the development of clinical practice guidelines, noted in this review as an organization/system level strategy that could help clinicians tailor care for immigrant and refugee women. In prior reviews, we also found a lack of conceptual guidance and research on what constitutes PCC for women in general [47, 48], and that guidelines lacked information on PCC or women’s health [49].

The strengths of this study included use of rigorous scoping review methods [22, 23], compliance with standards for the conduct and reporting of reviews [24] and use of a framework PCC to characterize facilitators and barriers [20]. Several issues may limit the interpretation and application of the findings. Despite having conducted a comprehensive search of multiple databases that complied with standards for search strategies [25] it was limited to English language studies. We did not search the grey literature given the methodological challenges that have been identified by others [50, 51]. The search strategy may not have identified all relevant studies or our screening criteria may have been too stringent. Few studies were eligible and those studies provided few specific details about PCC strategies or interventions. Risk of bias of included studies was not assessed as this is not customary for a scoping review [22, 23]. Although scoping reviews often include consultation with stakeholders to interpret the findings [22, 23], this step was not done because studies were few, and this study was one part of a larger investigation that has yet to be completed. Most studies addressed migrants in general and addressed multiple cultures from 24+ countries, so the findings appear to be transferrable. However, studies did not report findings by culture and the variety of cultures differed by study, so we lack insight on whether and how barriers and facilitators differ across groups that differ by culture, country of origin or religion.

## Conclusion

While few studies were included, we identified numerous determinants of high quality care for migrants; distinguished patient, clinician and organization/system level determinants; revealed aspects of care important to women migrants; and outlined a range of approaches and processes that can be employed to improve PCC for migrants from 24+ countries of origin, and in particular, women migrants. Barriers are facilitators were similar by type of migrant (refugees versus immigrants), care (general/primary, mental health, maternity), and research design (qualitative versus quantitative). Still, the few facilitators identified pertained largely to 2 PCC domains, thereby identifying gaps in knowledge of how to achieve PCC in 4 domains. As only 5 studies focused on migrant women, and no other studies reported sub-analyses by gender, we revealed a paucity of knowledge on how to achieve PCC for migrant women. Also, studies did not report findings by culture. Thus, further research is needed on determinants of, and interventions to support PCC for migrants that differ by culture, country of origin or religion, and particularly for migrant women.

## Supplementary information

**Additional file 1.** Search strategy. Strategy used to search databases for relevant studies.

**Additional file 2.** Data extracted from included studies. Table of data on study characteristics and findings.

**Additional file 3.** Comparison of facilitators and barriers by migrant status, type of care and study design.

## Data Availability

All data generated or analysed during this study are included in this published article [and its supplementary information files].

## Abbreviations

PCC: Patient-centred care
WHO: World Health Organization
PRISMA-Scr: Preferred Reporting Items for Systematic Reviews and Meta-Analysis criteria for scoping reviews
PICO: Population, Issues, Comparisons and Outcomes framework
