# Supplementary material for: Barriers and facilitators of patient centered care for immigrant and refugee women: a scoping review
Source: BMC Public Health. 2020 Jun 26;20:1013. doi: 10.1186/s12889-020-09159-6 (PMC7318468; doi:10.1186/s12889-020-09159-6)
Supplement: Supplementary file 1 — Additional file 1. Search strategy. Strategy used to search databases for relevant studies. [file 12889_2020_9159_MOESM1_ESM.docx]

Additional File 2. MEDLINE search strategy

| Search Statement | Results |
| --- | --- |
| Refugees/ | 9186 |
| "Emigrants and Immigrants"/ | 10643 |
| (asylum seekers or refugees or immigrants or newcomers).mp. | 32162 |
| 1 or 2 or 3 | 32162 |
| (patient centered or patient centred or patient-centered or patient-centred).mp. | 30604 |
| (family centered or family centred or family-centered or family-centred).mp. | 4053 |
| (client centered or client centred or client-centered or client-centred).mp. | 1401 |
| (person centered or person centred or person-centered or person-centred).mp. | 4711 |
| (woman centered or woman centred or woman-centered or woman-centred).mp. | 311 |
| Patient-Centered Care/ | 17321 |
| 5 or 6 or 7 or 8 or 9 or 10 | 38522 |
| 4 and 11 | 133 |
| limit 12 to (english language and yr="2010 -Current") | 101 |
| limit 13 to (case reports or editorial or interview or lecture or legal case or letter or news) | 3 |
| 13 not 14 | 98 |
