# Supplementary material for: Barriers and facilitators of patient centered care for immigrant and refugee women: a scoping review
Source: BMC Public Health. 2020 Jun 26;20:1013. doi: 10.1186/s12889-020-09159-6 (PMC7318468; doi:10.1186/s12889-020-09159-6)
Supplement: Supplementary file 2 — Additional file 2. Data extracted from included studies. Table of data on study characteristics and findings. [file 12889_2020_9159_MOESM2_ESM.docx]

Additional File 1. Data extracted from included studies

| Study | Objective | Research design | Population | Women-specific | Results |
| --- | --- | --- | --- | --- | --- |
| Harding  2019  Australia | Attitudes to and impact of treating refugees among general practitioners | Qualitative  Interviews with 14 general practitioners | Syria, Iraq, Sudan, Burma | No | Barriers:  Refugees have different expectations and needs:   - Refugees may have different expectations of the healthcare system; for example, the doctor has all the answers, doctor should be able to fix things, and bypass normal processes to “do it other ways” - May have different ways of viewing disease/illness; for example, seek help only when really sick, particularly need information on preventive health (i.e. screening) - Some unaware of time issues (i.e. need to be on time for appointments)   Impact on general practitioners:   - Helped broaden scope of practice and greater awareness of problems such as hepatitis and tuberculosis; however, for others, this was difficult and prompted feelings of anxiety - Addressing social and psychological issues represented a greater problem than dealing with other medical issues, requiring empathy - Support of individuals in distress prompted feelings of trauma and burnout among general practitioners - Those that were not confident in dealing with refugee-specific issues reached out to specialists and community resources   Impact on practice:   - Increased time needed for assessment, challenging in very busy practice - Language issues imposed barriers on communication - Some used telephone interpreters but this had limitations: interpreters can act as information gatekeepers and bring their own beliefs to the consultation, particularly problematic for addressing psychological issues - Using relatives for translation also problematic due to privacy and ethical implications, but used more frequently than telephone interpreters |
| Winn  2018  Canada | Barriers and facilitators of caring for pregnant refugee women | Qualitative  Interviews with 10 family physicians, nurses and social workers | Syria, Eritrea, Iraq, Congo, Afghanistan | Yes | Barriers:   - Diverse characteristics and migratory experience - Language - Cultural expectations and norms (i.e. not agreeing with labour induction, caesarean deliveries, use of epidurals) - Lack of resources to provide tangible support - Difficulty navigating complex system - Lack of familiarity with refugee health care needs/lack of training in this area   Facilitators:   - Team-based approach - Deliberate and clear communication - Take extra time to ensure patient’s comprehension - Coordination of multidisciplinary care within and outside of the clinic including lab appointments, specialists appointments, etc. - Build rapport with patients; better able to tailor care for individual - Dedication to caring and advocating for refugees |
| Mollah  2018  Australia | Barriers and facilitators of mental health care for immigrants and refugees | Qualitative    Interviews with 20 nurses, psychiatrists and counsellors | General | No | Barriers   - Tension between being culturally competent versus generalizing/stereotyping - Lack of awareness of how culture influences description of symptoms, presentation of illness, discussion of illness, diagnosis and treatment - Use of language services seen as time consuming, labour intensive, and sometimes inaccurate; many unfamiliar on how to use language services - Lack of support services in rural/remote communities - Western model of health care itself a cultural construct, inflexible, making it difficult to work with patients from outside that medical paradigm   Facilitators:   - Flexibility: open approach to each patient’s cultural background; recognize and accommodate cultural identity or language - Promote a culture of valuing diversity - Self-awareness of one’s own culture as influencing the therapeutic relationship - Professional development to have “working” knowledge of different ethnic backgrounds - Build rapport: careful navigation of numerous interpersonal and intercultural issues - Access to multilingual colleagues or interpreter service - Collaboration with community agencies - Personal dedication of health care professionals to have empathy and advocate for migrants |
| Murray  2018  Australia | Facilitators of medication management for Bhutanese refugees | Qualitative  Focus groups with 17 Bhutanese refugees (17.6% women) and 13 health care professionals (physicians, nurses, counsellors, pharmacists, social workers) | Bhutan | No | Facilitators:   - Provide reassurance about diagnosis and treatment that acknowledge cultural beliefs - Check understanding - Involve the person’s support network - Rely less on written and more on verbal or audiovisual instruction - Don’t rely on translated material as many have limited literacy in their own language - Continuity of clinicians and pharmacists; this would help to build rapport - Teamwork between providers to coordinate care - Use a bicultural worker to take them on a “tour” of relevant health services to orient them to the local healthcare system - Become familiar with the patient’s cultural background and migratory journey - Use skilled interpreters |
| Hjörleifsson  2018  Norway | Facilitators of caring for immigrants | Qualitative  Focus groups with 28 general practitioners | Asia, South America, Europe | No | Facilitators:   - Demonstrate knowledge and respect for their culture to help patients “open up” - Be aware of circumstances in their country of origin and migratory journey to inform appropriate enquires about potential trauma - Don’t generalize individuals according to their country of origin; really listen to the patient’s particular circumstances - Provide detailed information about health care services and procedures - Devote more time for consultations and/or divide tasks into multiple consultations - Use interpreters; professional translators preferred over family members - Ensure the patient accepts use of an interpreter - Take the initiative to organize interdisciplinary care/community support |
| Jones  2018  United States | Barriers and facilitators of rapport between inpatients and nurses | Qualitative  Interviews with 20 patients (65% women) | Mexico | No | Barriers   - Having needs of the nurse made them feel vulnerable - Felt like they were bothering the nurse if they had to call for assistance more than once - Communication difficult due to language barriers, leading to uncertainty among patients and nurses   Facilitators   - Use of translators or hand gestures helped communication - Struggle to understand each other led to bond formation between patient and nurse - Greeting the patient and taking the time to chat informally helped bonding - Chatting was appreciated even when the nurse did not speak the patient’s language and the patient was unable to respond; speaking English was better than not speaking at all - The nurse’s “way of being” included:   - emotional aspect of caring about the patient, reflecting personality and attitude: kind, open, friendly, positive   - physical aspect of caring for the patient: willing to help, physically present, spent time with patient to do their job to fulfill the patient’s needs, not just to complete their tasks - All these facilitators fostered trust - Trust helped patient feel more comfortable, relaxed, safe, supportive, encouraged, willing to try new things - Trust was possible even when language a barrier when nurse was perceived as caring for the patient well, which was based on nurses way of being (emotional, physical) |
| Mohammadi  2017  Sweden | Barriers of maternity care among Afghan women immigrants | Qualitative  Interviews with 11 afghan women who recovered from near miss morbidity during childbirth, and 4 husbands | Afghanistan | Yes | Barriers:   - Language: women could understand little of what clinicians advised them, were embarrassed to ask questions - Poor communication: clinicians were busy and no time for careful interaction - They also dismissed complaints, delaying attention to concerns - Physicians did not recognize problems in a timely manner, or they were misdiagnosed - Attended consultations for antenatal care but received inadequate information about potential complications - Economic constraints and lack of health insurance were a barrier to timely seeking and accessing care services - Some women said that decisions their parents-in-law made were barriers of seeking care - Overt discrimination, disrespectful behaviour, disparaging remarks made by clinicians - Provided with little information about the near miss incidents, reasons they developed complications |
| Paternotte  2017  Netherlands | Facilitators of communication for “non-native” patients | Qualitative  Interviews with 30 non-native patients sampled from gynaecology, internal medicine, urology and orthopedic surgery outpatient clinics (% women not reported) | Surinam, Turkey, Morocco, Portugal, Indonesia, Iraq, China, Ireland | No | Facilitators:   - Doctor characteristics: ethnic background not important so long as they were professional; many preferred doctor of same gender; experienced shame with doctor of opposite gender; some expressed preference for older doctors who were considered more trustworthy - Communication: speak slowly, use short sentences, explain topics in various ways, avoid medical jargon, listen to patient, take sufficient time - Interaction: comfort the patient, focus attention on patient rather than computer, prepare for the consultation ahead of time, honest about diagnosis, welcome the patient upon meeting, treat patient as unique and not as a disease, acknowledge concerns expressed by patient, ask questions to develop shared understanding - Most patients thought it was necessary for doctor to ask questions about cultural background; some were afraid that doctors would then make assumptions about them - Those with language challenges preferred an informal rather than professional interpreter - Important to provide information about the local health care system - A good doctor-patient relationship (rapport) essential for satisfactory communication; established over period of time |
| Larsson  2016  Sweden | Experience of providing care to immigrant women seeking abortion | Qualitative  Interviews with 3 doctors and 10 midwives at abortion clinics | General | Yes | Barriers:   - Women have little knowledge about female anatomy, menstrual cycle, reproduction, contraceptives - Lack of knowledge leads to misunderstanding and miscommunication with clinicians - Cultural background and religious beliefs influence decisions about contraceptive use leading to unplanned pregnancies and repeat abortions - Sometimes rely on opinions of a male partner or female family or friends rather than clinician to make decisions about contraceptive use or method - Male partners also lacked knowledge about contraceptives and required educational counseling - Consultations take more time due to language, culture and knowledge barriers - Remuneration insufficient for amount of time required - Fear of their families finding out about pregnancy/abortion (honour-based violence) - Fear of being caught taking contraceptives (preferred methods that could be hidden such as IUD or hormone implant) - No protocols of guidelines to help clinicians care for immigrant women |
| Paternotte  2016  Netherlands | Barriers of caring for “non-native” patients | Qualitative  Interviews with 17 specialists (gynecology, internal medicine, orthopedic surgery) after viewing 2 videos of consultation with native and non-native patient | Morocco, Turkey, Nicaragua, Hungary, Australia, Belgium, Pakistan, Nigeria | No | Barriers   - Initially did not identify different communication styles; emphasized that it depended by individual patient characteristics such as education level or language limitations rather than cultural background, and said they adapted to suit the patient - Did not recognize that culture could influence patient’s communication style - Found it difficult to identify expectations of patients with different cultural backgrounds - Did not always explicitly ask patients about reason for consultation or specific problems they wanted to discuss - Perceived that patients wanted the doctor to lead the conversation, leading to a directive style of communication - Language differences can lead to misunderstandings - Using interpreters was time-consuming and challenging - Preferred family members rather than formal interpreters - Took greater effort to put non-native patients at ease - Lacked undergraduate or postgraduate training |
| Phillippi  2016  United States | Facilitators of prenatal care for immigrant women at a nurse-led clinic | Qualitative  Interviews with 50 immigrant women | Cambodia, Somalia, Mexico, South America, Burma, Iraq, Syria | Yes | Facilitators:   - Connection (rapport) was an overriding theme; women were willing to overcome barriers if they knew they would be treated as a unique and special person - Thus, clinics needed to be flexible to women’s needs, culture and beliefs - Early experience with clinic also mattered (i.e. ease of first phone call, getting appointment soon after) - Often interested in a less medical approach if more compatible with religious or cultural beliefs - Preferred women clinicians - Valued relaxed atmosphere; conducive to communication - Staff and clinicians who conveyed a caring attitude - Time to ask questions or express concerns without being judged as uneducated - Appreciated being provided with information to be involved in decision-making |
| Clochesy  2015  United States | Barriers of care among immigrants | Qualitative  Focus Groups with 60 patients (46.7% women, 31.7% were immigrants) | Mexico, South America, Russia | No | Barriers   - Delay in diagnosis - Feeling rushed by clinician - Red tape (policies, programs) or paperwork - Feeling more like a lab rat than a person - Perception of being judged - Feels like they are treated differently due to culture, race, gender, etc. - Desired easier system navigation, to be involved in decisions, privacy - Wanted clinicians to care about them, listen, helps them understand, gets to know them, articulates goals for the consultation and for the patient |
| De Jesus  2014  United States | Facilitators of mental health care among immigrants | Qualitative  Focus groups with 48 patients (50% women) | Brazil or Cape Verde (Portuguese-speaking) | No | Facilitators:   - Clinicians are attentive, respectful, non-judgmental, encouraging, professional - Clinician communicates in a comprehensible manner, explains instructions clearly - Clinician is familiar with patient’s cultural background, is aware of cultural differences |
| Papic  2012  Canada | Barriers and facilitators of care for immigrants | Quantitative  Questionnaire of family physicians (598, 44.8% responded) | General | No | Barriers:   - Communication due to language and culture differences - Compliance with treatment recommendations - Most used informal rather than professional interpreters; most believed it was the patient’s responsibility to make such arrangements (not their own) - Lacked cross-cultural training (medical or continuing) - Lack of monetary incentives to care for immigrants, which they perceived as more challenging than caring for non-immigrant patients   Facilitators:   - Learned a few words of the language - Offered print material in various languages - Booked longer appointments - Used visual cues - Used repetition |
| Hasnain  2011  United States | Barriers and facilitators of caring of immigrant Muslim women | Quantitative  Questionnaire of 80 clinicians (physicians, nurses, allied health) and 27 women (59% immigrants) | Muslim women | Yes | Facilitators:   - Clinician communication skills, attitudes, and gender, ethnicity or religion (both patients and clinicians agreed) - Patient communication skills (both patients and clinicians agreed) - Clinician competency and availability (patients only) - Patient attitudes (clinician only)   Barriers:   - Clinician lacks understanding of patient’s culture/religion (both) - System issues such as insurance, transportation (both) - Patients lack of trust of health care system (both) - Patients prefer woman clinician (clinicians only) - Patients lack knowledge about disease processes (clinicians only) |
| Lo  2010  United States | Barriers of caring for immigrants | Qualitative  Interviews with 24 physicians (family, internal medicine) | General | No | Barriers   - Lack of knowledge about other cultures - Lack of training on how to initiate an in-depth conversation to understand how culture may have been contributing to the medical concern - Lack of time to thoroughly explore these issues - Immigrants brought expectations based on cultural norms - Some also wanted doctor to make decisions, which contradicts patient autonomy - Unclear how to deliver medical care while accommodating cultural norms - Participants described this as “cultural translation” and they were challenged to hybridize the Western medical model with the patient’s cultural model - Some patients were alienated from the medical professional through past negative experiences, which influenced their willingness to comply with testing or treatment - Long-term relationships were needed to eventually piece together a patient’s life context, expectations and personal sense-making schemas, emphasizing the need for continuity - Some patients felt shame for their life condition and were reluctant to discuss them - Time constraints, lack of interpreter services exacerbated language barriers |
