# Supplementary material for: Barriers and facilitators of patient centered care for immigrant and refugee women: a scoping review
Source: BMC Public Health. 2020 Jun 26;20:1013. doi: 10.1186/s12889-020-09159-6 (PMC7318468; doi:10.1186/s12889-020-09159-6)
Supplement: Supplementary file 3 — Additional file 3. Comparison of facilitators and barriers by migrant status, type of care and study design. [file 12889_2020_9159_MOESM3_ESM.docx]

Additional File 3. Comparison of facilitators and barriers by migrant status, type of care and study design

| Study | Facilitators | Barriers |
| --- | --- | --- |
| REFUGEES |  |  |
| Harding [26]  2019  Australia  General care  Clinician participants | --- | - Have different expectations of healthcare system - Have different views of disease - Addressing social/psychological problems - Dealing with their trauma results in burnout - Increased time for assessment - Language barriers - Formal and family interpreters both have limitations |
| Winn [27]  2018  Canada    Maternity care  Patient participants  Specific to women | - Team approach - Deliberate/clear communication - Take extra time - Coordinate multidisciplinary care - Build rapport - Must be dedicated to their care | - Diverse migratory experience - Language - Cultural expectations/norms - Navigating healthcare system - Clinicians lack training |
| Murray [29]  2018  Australia    General care  Patient/clinician participants  Specific to Bhutan | - Acknowledge culture/beliefs - Check understanding - Involve their support network - Rely less on written and more on verbal or audiovisual instruction - Don’t rely on translated material as many have limited literacy in their own language - Build rapport thru continuity - Teamwork to coordinate care - Use a bicultural worker to take them on a “tour” of health services - Become familiar with culture and migratory journey - Use skilled interpreters | --- |
| Mohammadi [32]  2017  Sweden    Maternity care  Patient participants  Specific to women  Specific to Afghanistan | --- | - Language – embarrassed to ask questions - Clinicians lack time for communication - Disrespectful behaviour - Clinicians dismiss concerns and miss diagnosing problems in timely way - Provide inadequate information - Economic constraints and lack of health insurance influence access to health services - Decisions driven by parents-in-law |
| IMMIGRANTS |  |  |
| Hjörleifsson [30]  2018  Norway    General care  Clinician participants | - Demonstrate knowledge and respect for their culture - Learn about country and migratory journey - Don’t generalize individuals according to their country of origin - Provide detailed information about health care services and procedures - Devote more time for consultations Use interpreters; formal preferred - Ensure patient accepts use of interpreter | --- |
| Jones [31]  2018  United States  Inpatient care  Patient participants  Specific to Mexico | - Greet patients, chat informally - Use translators - Friendly personality of nurse | - Feel vulnerable to rely on nurse - Language |
| Larsson [34]  2016  Sweden    Abortion care  Clinician participants  Specific to women | --- | - Women know little about anatomy or reproduction - Decisions about contraception based on culture – unplanned pregnancies, repeat abortions - Fear retribution from family if contraception used - Influenced by male partner/in-laws, who also lacked knowledge - Consultations take more time - Remuneration insufficient for time - No protocols or guidelines |
| Phillippi [36]  2016  United States    Maternity care  Patient participants  Specific to women | - Establish rapport - Understand cultural needs - Women clinicians - Friendly staff and clinicians - Time to ask questions/concerns - Lack of judgment - Information to support shared decisions | --- |
| De Jesus [38]  2014  United States  Mental health care  Patient participants | - Clinicians are respectful and non-judgmental - Communicate clearly - Familiar with culture/beliefs | --- |
| Papic [39]  2012  Canada    General care  Clinician participants | - Learned a few words of language - Offer print info in their language - Longer appointments - Use visual cues and repetition | - Language and culture differences - Compliance with treatment - Lack of cross-cultural training - Lack of monetary incentives |
| Hasnain [40]  2011  United States  General care  Patient/Clinician participants  Specific to women | - Clinician communication skills, gender, ethnicity or religion - Patient communication skills - Clinician competency (patients) - Patient attitudes (clinicians) | - Clinicians lack awareness culture/religion - Patients lack insurance, transportation - Patients don’t trust healthcare systems - Patients prefer woman clinician - Patients lack knowledge of disease processes (clinicians) |
| Lo [41]  2010  United States  General care  Clinician participants | --- | - Lack knowledge about other cultures - Lack of training - Lack of time - Language barriers/lack of interpreters - Different expectations based on culture - Some also wanted doctor to make decisions, which contradicts patient autonomy - Patient compliance with treatment - Patients reluctant to discuss prior life |
